# Transcriptomic Responses to Different Cry1Ac Selection Stresses in *Helicoverpa armigera*

**DOI:** 10.3389/fphys.2018.01653

**Published:** 2018-11-22

**Authors:** Jizhen Wei, Shuo Yang, Lin Chen, Xiaoguang Liu, Mengfang Du, Shiheng An, Gemei Liang

**Affiliations:** ^1^State Key Laboratory of Wheat and Maize Crop Science, College of Plant Protection, Henan Agricultural University, Zhengzhou, China; ^2^State Key Laboratory for Biology of Plant Diseases and Insect Pests, Institute of Plant Protection, Chinese Academy of Agricultural Sciences, Beijing, China

**Keywords:** *Helicoverpa armigera*, DGE, Cry1Ac, trypsin, receptors, mechanisms of resistance

## Abstract

*Helicoverpa armigera* can develop resistance to *Bacillus thuringiensis* (Bt), which threaten the long-term success of Bt crops. In the present study, RNAseq was employed to investigate the midgut genes response to strains with different levels of resistance (LF5, LF10, LF20, LF30, LF60, and LF120) in *H. armigera*. Results revealed that a series of differentially expressed unigenes (DEGs) were expressed significantly in resistant strains compared with the LF-susceptible strain. Nine trypsin genes, *ALP2*, were downregulated significantly in all the six resistant strains and further verified by qRT-PCR, indicating that these genes may be used as markers to monitor and manage pest resistance in transgenic crops. Most importantly, the differences in DEG functions in the different resistant strains revealed that different resistance mechanisms may develop during the evolution of resistance. The immune and detoxification processes appear to be associated with the low-level resistance (LF5 strain). Metabolic process-related macromolecules possibly lead to resistance to Cry1Ac in the LF10 and LF20 strains. The DEGs involved in the “proton-transporting V-type ATPase complex” and the “proton-transporting two-sector ATPase complex” were significantly expressed in the LF30 strain, probably causing resistance to Cry1Ac in the LF30 strain. The DEGs involved in binding and iron ion homeostasis appear to lead to high-level resistance in the LF60 and LF120 strains, respectively. The multiple genes and different pathways seem to be involved in Cry1Ac resistance depending on the levels of resistance. Although the mechanisms of resistance are very complex in *H. armigera*, a main pathway seemingly exists, which contributes to resistance in each level of resistant strain. Altogether, the findings in the current study provide a transcriptome-based foundation for identifying the functional genes involved in Cry1Ac resistance in *H. armigera*.

## Introduction

The Cry1Ac toxin from *Bacillus thuringiensis* (Bt) is harmless to most organisms and considered as an environmentally friendly pesticide. Hence, Cry1Ac toxin is used commercially as a bio-insecticide and expressed in transgenic plants for controlling insect pests (Wu et al., 2008; Hutchison et al., 2010; Edgerton et al., 2012; Lu et al., 2012; Klümper and Qaim, 2014). The area of Bt transgenic crops planted worldwide has rapidly increased to 98.5 million hectares in 2016 and has accumulated more than 830 million hectares since 1996 (James, 2016). Although Bt crops have brought great economic and environmental benefits (Wu et al., 2008; Hutchison et al., 2010; Edgerton et al., 2012; Lu et al., 2012; Klümper and Qaim, 2014), the development of resistance to Bt toxins can reduce or even eliminate these benefits (Tabashnik et al., 2013; Van den Berg et al., 2013; Farias et al., 2014; Gassmann et al., 2014; Tabashnik and Carrière, 2017). Unfortunately, the cumulative number of cases of practical resistance to the Bt toxins in transgenic crops surged from 3 in 2005 to 16 in 2016 according to a report from Tabashnik and Carrière (2017) attracting researcher's attention.

Understanding the mode of Bt action and the mechanisms that confer resistance to Bt toxins can help to sustain and even enhance their efficacy to control pests. Models of Bt action agree that Bt protoxins are first converted to activated toxins by insect midgut proteases and then the activated toxins bind to the insect midgut receptors, finally leading to insect death (Gill et al., 1992; Pardo-López et al., 2013; Adang et al., 2014). Four main functional receptors of the Cry1Ac toxin have been identified and verified from the brush border epithelium, including alkaline phosphatase (ALP) (Flores-Escobar et al., 2013), cadherin (Chen et al., 2014), aminopeptidase (APN) (Zhang et al., 2009; Tiewsiri and Wang, 2011; Valaitis, 2011; Flores-Escobar et al., 2013; Wei et al., 2016b), and ATP-binding cassette transporter proteins (ABCs) (Tanaka et al., 2017). The identification of Cry1A receptors has broadened our understanding of Cry1Ac action. However, the mode of action of Cry1Ac is very complex and a change in any step of the toxicology process will inevitably lead to insect resistance. The most common Bt-resistant mechanisms had been reported in Lepidoptera, including a reduced binding capacity of Bt toxins to midgut receptors by a decrease in the activity and transcription of ALP or APN as well as mutations of APN, cadherin, and ABCC_2_ (Xu et al., 2005; Zhang et al., 2009, 2012; Gahan et al., 2010; Baxter et al., 2011; Jurat-Fuentes et al., 2011; Atsumi et al., 2012; Xiao et al., 2014; Chen et al., 2015) and a reduced conversion of the protoxin to the toxin by downregulation of trypsin (Rajagopal et al., 2009; Cao et al., 2013; Liu et al., 2014; Wei et al., 2016a). These studies indicate that many genes are involved in resistance of insects to Bt toxins. In addition, it sounds like it is hard to identify the common marker to monitor and manage pest resistance in transgenic crops.

Most importantly, different genes may show different contributions to resistance at different development levels. The decreasing protoxin activation in *Ostrinia nubilalis* (HD-1 Bt kurstaki-resistant strain) caused a 47-fold resistance to Dipel, which contained Cry1Ab, Cry1Aa, Cry1Ac, and Cry2Aa (Li et al., 2004, 2005). In a 100-fold more Cry1Ab-resistant *Diatraea saccharalis* strain, the resistance to the Cry1Ab toxin is due to the lower gene expression level of cadherin (Yang et al., 2011). In the *H. armigera* GYBT-resistant strain, a deletion between exons 8 and 25 of the cadherin gene resulted in a 564-fold resistance to Cry1Ac-activated toxin (Xu et al., 2005). For the Cry1Ac-resistant BtR strain, it was identified that a deletion mutation of APN3 and the downregulation of cadherin lead to Cry1Ac resistance. A subsequent study confirmed that a deletion mutant in the APN1 gene caused a more than 2,971-fold resistance to Cry1Ac in the BtR strain (Wang et al., 2005; Zhang et al., 2009). Recently, the mutations in the ABCC2 transporter and the cadherin genes were reported to have a different affect on the binding of Cry toxins to the proteins on the brush border membrane vesicle (BBMV) from *H. virescens* (Gahan et al., 2010). The mutations of the ABCC2 transporter showed a higher level of resistance to Cry toxins. These studies demonstrate that different genes have different impacts on resistance levels.

Moreover, the resistant pathways were studied also to find the key factors that regulated the expression of resistant genes. Guo et al. (2015b) reported a novel transregulatory signaling mechanism in which the mitogen-activated protein kinase (MAPK) signaling pathway was confirmed to be responsible for regulating the expressions of ALP and ABCC genes in a field-evolved resistant strain of *P. xylostella*. Also, the constitutively transcriptionally activated upstream gene, MAP4K4, in the MAPK signaling pathway is responsible for this transregulatory signaling mechanism (Guo et al., 2015b). Importantly, the key resistant factor, MAP4K4, may be used for molecular control of the Cry1Ac resistance.

However, the genes and pathways affected the Cry1Ac resistance depending on the Cry1Ac selection stresses in *H. armigera* have not been comprehensively assessed. The next-generation DNA sequencing provides a research technique to study the changes in gene expression in the midgut transcriptomes of Bt-resistant and -susceptible strains; this technology can detect differentially expressed genes in biochemical pathways involved in Bt resistance and provide new insights into resistance mechanisms (Lei et al., 2014; Nanoth Vellichirammal et al., 2015; Zhang et al., 2017). In this study, a susceptible LF (a laboratory strain collected from Langfang) strain and six resistant LF strains were selected for an analysis of related resistance genes, in particular, six substrains of LF came from the LF strain by selecting a series of gradually increasing resistant strains (Cao et al., 2013, 2014; Liu et al., 2014; Xiao et al., 2014; Chen et al., 2015; Wei et al., 2016a). First, RNA sequencing was employed to construct a complete and comprehensive reference transcriptome database from midgut samples of these seven strains. The differentially expressed genes were detected further among these seven strains by digital gene expression analysis (DGE). These data provide a foundation for understanding the systemic differences between Cry1Ac-resistant strains and Cry1Ac-susceptible strain and might aid in finding candidate resistance. Most importantly, the analysis of differently expressed genes among the seven strains will uncover the role of different genes in the different resistance phases and might explain how selection can cause fixed changes of the expression levels of numerous genes.

## Materials and methods

### Insect strains

The LF-susceptible H. armigera strain was established from a field population by collecting from the Langfang County, Hebei Province of China in 1998. The LF strain was reared in a lab environment without exposure to any insecticides (Wu and Guo, 2004). The six LF substrains came from the LF-susceptible strain via a series of selections: LF5, LF10, LF20, LF30, LF60, and LF120 (Cao et al., 2013, 2014; Liu et al., 2014; Xiao et al., 2014; Chen et al., 2015; Wei et al., 2016a; Table 1). The strain name is in accordance to the selection concentration for each strain, where the number from 5 to 120 follows LF: such as LF5 was selected with a 5 μg/ml Cry1Ac protoxin artificial diet (Liang et al., 2008). In this study, LF5, LF10, LF20, LF30, LF60, and LF120 had been selected for 60, 52, 42, 38, 21, and 17 generations with corresponding Cry1Ac diets, respectively (Table 1).

**Table 1:** Responses to Cry1Ac of the susceptible strain (LF) and six resistant strains (LF5, LF10, LF20, LF30, LF60, and LF120) of *H. armigera*.

**Strains**	**Dose of selection (μg/ml)**	**Gen^a^**	**LC_50_(95%, FL)^b^ μg/cm^2^**	**RR^c^**
LF	0	99	0.0270 (0.011–0.052)	1.0
LF5	5	60	14.6 (7.4–31)	540
LF10	10	52	17.3 (6.0–63)	640
LF20	20	42	23.0 (12–47)	850
LF30	30	38	27.8 (9.9–56)	1,000
LF60	60	21	28.6 (19–41)	1,000
LF120	120	17	54.2 (31–100)	2,000

a *Generation*.

b *Concentration killing 50% with 95% fiducial limits in parentheses, units are μg toxin per cm^2^ diet*.

c *Resistance ratio, the LC_50_ for a strain divided by the LC_50_ for LF*.

### Bioassay of resistance to Cry1Ac toxin

Larval responses to Cry1Ac toxin were evaluated using the methods reported by Wei et al. (2015). The Cry1Ac protoxin crystals were obtained from the HD-73 strain of *B. thuringiensis* (kindly supplied by Biotechnology Research Group, Institute of Plant Protection, Chinese Academy of Agricultural Sciences). Totally, 72 neonates per concentration for each treatment were tested. About 7 days later, dead insects and those that were still first instars were scored as dead. Five or more toxin concentrations were used to calculate the LC_50_ values of each strain (Table 1).

### Dissection of midgut and extraction of RNA

Larvae from different colonies (LF, LF5, LF10, LF20, LF30, LF60, and LF120) were reared with a non-Bt toxin diet under standard rearing conditions. The midgut tissues of larvae in fifth instars (*n* = 30 per pool) were dissected from different strains. The lumen was then rapidly washed with a solution of 0.7% NaCl (w/v) to remove debris. Two biological replicates were employed. Total RNA from every replicate was extracted separately from each pool (LF, LF5, LF10, LF20, LF30, LF60, and LF120) using the Trizol reagent according to manufacturer's suggestions (Invitrogen, CA). In order to remove genomic DNA contamination, resulting RNA was treated with DNase I (Promega, Madison, WI, USA) following manufacturer's instructions. Quantity and quality of total RNA were assessed by denaturing gel electrophoresis and spectrophotometry on a Nanodrop 2000 (Thermo Scientific, Wilmington, DE, USA).

### Preparation of library for analysis of transcriptome

Sequencing libraries were generated from 3 μg total RNA per sample using NEBNext® Ultra™ Directional RNA Library Prep Kit for Illumina® (NEB, USA) following manufacturer's recommendations. Briefly, mRNA was separated from total RNA using poly-T oligo-attached magnetic beads. First-strand cDNA was generated using random hexamer primer and M-MuLV Reverse Transcriptase (RNaseH-) followed by second-strand cDNA synthesis using DNA Polymerase I and RNase H. Remaining overhangs were converted into blunt ends via exonuclease/polymerase activities. The 3′ ends of DNA fragments were firstly adenylated and then NEBNext Adaptors with hairpin loop structure were ligated to prepare for hybridization. To select cDNA fragments of preferentially 150–200 bp in length, the library fragments were purified with AMPure XP system (Beckman Coulter, Beverly, USA). About 3 μL USER Enzyme (NEB, USA) was used with size-selected, adaptor-ligated cDNA at 37°C for 15 min followed by 5 min at 95°C before PCR. Then PCR was performed with Phusion high-fidelity DNA polymerase, Universal PCR primers, and Index (X) Primer. The PCR products were purified finally (AMPure XP system) and the quality of library was assessed on the Agilent Bioanalyzer 2100 system.

### Analysis of results of illumina sequencing

The clustering of the index-coded samples was carried out on a cBot Cluster Generation System using TruSeq PE Cluster Kit v3-cBot-HS (Illumia) according to the manufacturer's instructions. After cluster generation, the library preparations were sequenced on an Illumina Hiseq 2000 platform and paired-end reads were generated. Transcriptome assembly was accomplished by using Trinity r20121005 (Grabherr et al., 2011) with min_kmer_cov set to 2 by default and all other parameters set default. Raw data (raw reads) of fastq format were firstly processed through in-house perl scripts. Clean data (clean reads) were then obtained by removing noise signals (reads containing adapter, reads containing ploy-N, and low-quality reads) from raw data. The following data analyses were performed based on the clean data. After eliminating redundancy using cd-hit and cap3 software, these data were mixed with our unpublic data of cotton bollworm transcriptome database. The resulting unigene database was used as a reference transcriptome database for subsequent analysis of DGE. The homology searches of all unigenes were performed based on BLASTx and BLASTn programs against the GenBank non-redundant protein (nr) and nucleotide sequence (nt) database at NCBI (v2.2.28). Matches of an *E*-value < 1.0E-5 were considered to be significant (Altschul et al., 1997). Gene ontology term (GO, http://www.geneontology.org/) annotations were assigned by Blast2GO software (b2g4pipe_v2.5) (Götz et al., 2008). The KOG (euKaryotic Ortholog Groups) and KEGG (Kyoto Encyclopedia of Genes and Genomes) annotations were performed using Blastall software against the KOG database (http://www.ncbi.nlm.nih.gov/COG/) and KEGG database (http://www.genome.jp/kegg/), respectively.

### DGE library preparation and sequencing

Library for DGE sequencing was prepared according to earlier-mentioned method (see “Library preparation for transcriptome analysis”). After cluster generation, the library sequencing was performed on an Illumina Hiseq 2000 platform and 100 bp single-end reads were generated.

### Analysis and mapping of DGE

After removing reads containing adapter, reads containing ploy-N, and low-quality reads from raw data, the clean data were then obtained. All analyses were performed according to the clean data. For unigene DEG, single-end clean reads were aligned to the unigene sequences by Bowtie v0.12.9. The HTSeq v0.5.4p3 was used to count unigene DEG numbers mapped to each unigene. Reads per kilobase of exon model per million mapped reads (RPKM) of each gene were calculated based on the length of the gene and reads count mapped to this gene (Mortazavi et al., 2008). The differentially expressed unigenes were used for mapping and annotation.

### Evaluation of DGE libraries

The frequency of each unigene in the different cDNA libraries was analyzed to compare gene expression in different strains. The DEGSeq R package (1.12.0) was used to analyze the differential expression of two conditions. The *P*-values were adjusted using the Benjamini & Hochberg method. Significant differential expression genes were obtained using set threshold values [corrected *P*-value of 0.005 and log_2_ (Fold-change) of 1]. For pathway-enrichment analysis, we mapped all the differentially expressed genes to terms in the GO data database and KEGG database. The GO-enrichment analysis of differentially expressed genes was implemented by the GOseq R package, in which gene length bias was corrected. The GO terms with corrected *P*-value < 0.05 were considered significantly enriched by differentially expressed genes. We used KOBAS software to test the statistical enrichment of differential expression genes in KEGG pathways.

### Validation of qRT-PCR

The first-strand cDNA of each strain was used as the template for real-time PCR analysis. Each strain of *H. armigera* included 90 larvae (30 larvae per biological replicate). The mRNA expression levels of ALP-like, ALP2, APN5, and APN1 in different strains were analyzed by a quantitative real-time PCR (qRT-PCR). The β-actin and GAPDH of *H. armigera* were used as internal reference genes (Liu et al., 2014). The primers of the said genes used for qRT-PCR analysis are listed in Table S1. Each qRT-PCR (TaqMan) (TIANGEN, FP206, China) reaction was performed individually in a 20-μL system containing 1 μL of the template cDNA, 10 μL of the 2 × SuperReal PreMix (Probe), 0.6 μL of the 10uM of each primer, 0.4 μL of the 10 uM of the probe, 0.2 μL of the 50 × ROX Reference Dye^*^3, and 7.2 μL of the RNase-Free ddH_2_O. The thermal cycler conditions used for real-time PCR were: 40 cycles of 3 s at 95°C and 30 s at 60°C. The mRNA expression levels of trypsin genes were tested by SYBR Green Supermix (TaKaRa). The primers of trypsin genes used for qRT-PCR analysis can be found in Table S2. The *H. armigera* 18S (Du et al., 2017) and EF1-α (Yuan et al., 2006) were used as internal reference genes. The qRT-PCR was performed at 95°C for 3 min, followed by 40 cycles of 95°C for 15 s and 60°C for 20 s. Real-time PCR of trypsin and reference genes was done in a 20-μL reaction system containing 10 μL of 2 × SYBR Mix and 10 μM forward primer and reverse primer (1.0 μL each), 1 μL template cDNA, and 7.0 μL nuclease-free water. All qRT-PCR reactions were performed in 96-well optical plates in an ABI 7500 Real-time PCR System (Applied Biosystems).

The expression levels of all the earlier-mentioned genes were calculated with their amplification efficiency (E) and mean Ct, and the expression levels of the candidate genes were normalized with the geometric mean of the expression of each of the two reference genes (GAPDH and EF-1α/18S and EF1-α; Livak and Schmittgen, 2001; Vandesompele et al., 2002; Liu et al., 2015). The results of each gene among different strains were determined with one-way analysis of variance (ANOVA), followed by Tukey's honestly significance difference (HSD) test for mean comparison. All statistical analysis was performed with SPSS v.18.0 (SPSS Inc., Chicago, IL, USA) at *P* < 0.05 level of significance.

## Results

### Insect resistance levels

After 60 generations of Cry1Ac selection, the LF5 laboratory colony had an estimated resistance ratio of 540 compared with the susceptible LF strain (Table 1). The LF10 was divided from LF5; then, after selection for 52 generations using diets containing 10-μg/mL Cry1Ac toxin, the resistance ratio of the LF10 strain cotton bollworm reached 640 for Cry1Ac toxin (Table 1). The LF20 was divided from LF10 and after selection for 42 generations on diets containing 20 μg/mL Cry1Ac toxin, the resistance ratio of LF20 strain cotton bollworm increased to 850. Similarly, LF30 was divided from LF20 and selected for 38 generations on diets containing 30-μg/mL Cry1Ac toxin; in addition, LF60 was divided from LF30 and selected for 21 generations on diets containing 60-μg/mL Cry1Ac toxin. The resistance ratio of these two strains was 1,000 when compared with the susceptible-LF strain (Table 1). The LF120 was divided from LF60 and selected for 17 generations on diets containing 120-μg/mL Cry1Ac toxin; the LF120 strain showed the highest resistance levels (2,000-fold) (Table 1). Generally, with the increase of the selection concentration of Cry1Ac toxin, the resistance levels were improved correspondingly (Table 1).

### Illumina sequencing and transcriptome assembly

In total, 77,422,352 clean reads were obtained from transcriptomics analysis of samples obtained from the midguts of the seven strains and were assembled into 66,502 transcripts. The mean length of the transcripts was 1,324 bp with lengths ranging from 201 to 49,954 bp. After mixing with our private cotton bollworm transcriptome database, a total of 139,012 unigenes were obtained. The size distribution of these unigenes is shown in Figure S1.

### Annotation of predicted proteins

Annotation of gene function was performed by running Blast on the following databases: Nr (NCBI non-redundant protein sequences), Pfam (Protein family), Nt (NCBI non-redundant nucleotide sequences), Swiss-Prot (A manually annotated and reviewed protein sequence database), KOG (euKaryotic Ortholog Groups), GO (Gene Ontology), and KO (KEGG Ortholog database). The results demonstrated that 64.96% unigenes were annotated in NR, 35.14% were annotated in NT, 15.83% were annotated in KO, 43.77% were annotated in SwissPort, 41.29% were annotated in PFAM, 48.11% were annotated in GO, and 32.96% were annotated in KOG. In total, 6.12% unigenes were annotated in all Databases. Finally, most of the 139,012 unigenes (72.89%) were matched to known genes. This transcriptome database will be used as a reference database to analyze differences in gene expression among different strains of cotton bollworm.

### Classification of gene ontology (GO)

**Figure 1:**
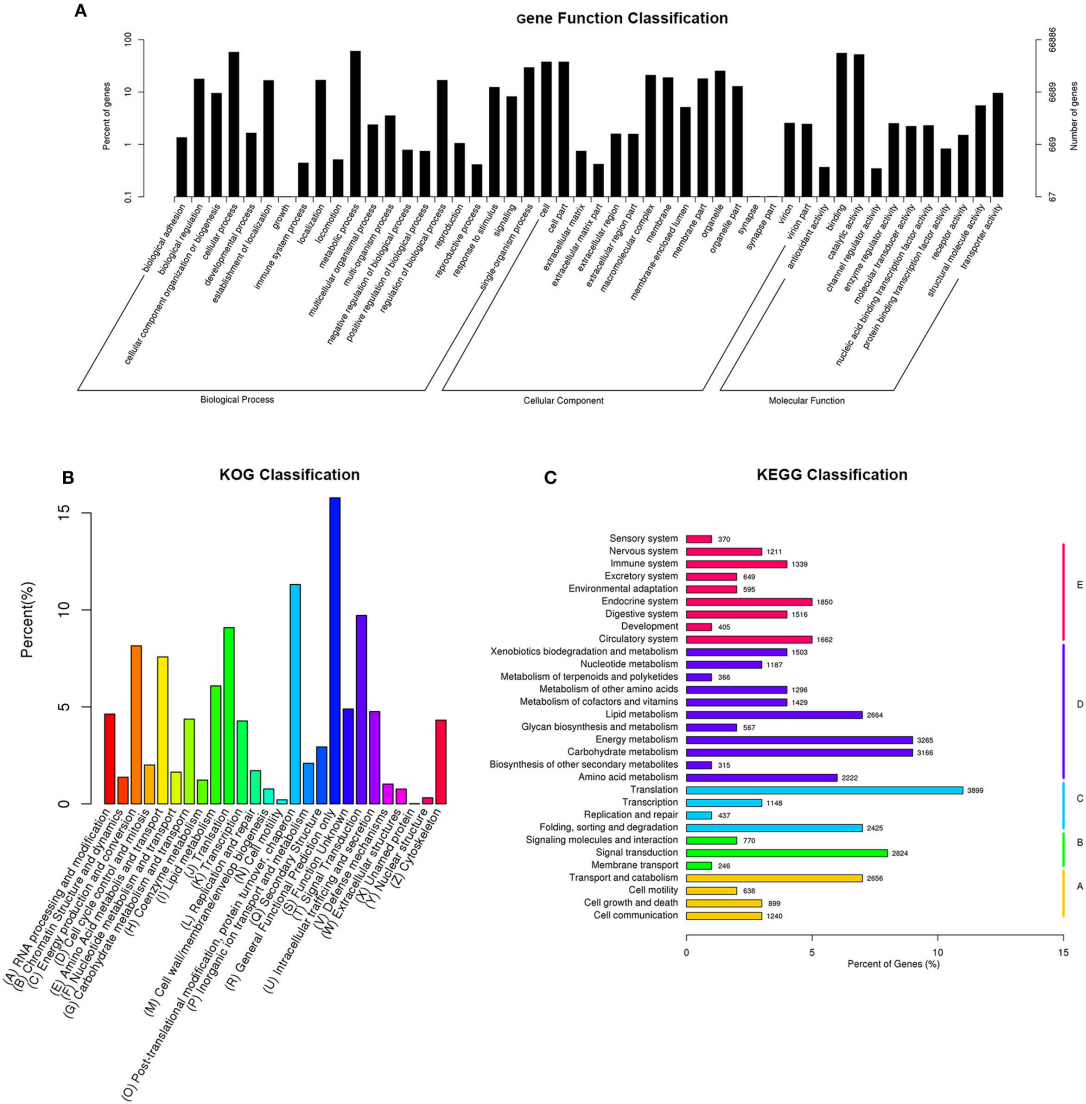
Histogram of gene ontology classification **(A)**, Clusters of Orthologous Groups of proteins **(B)**, and KEGG Ortholog database **(C)**. **(A)** The results are summarized in three main categories: biological process, cellular Component, and molecular function. The right y-axis indicates the number of genes in a category. The left y-axis indicates the percentage of a specific category of genes in that main category. **(B)** The X-axis indicates 26 group categories; the Y-axis indicates the percentage of a specific category of genes in that main category. **(C)** The Y-axis is the enrichment of the KEGG term, the X-axis indicates the percentage of a specific category of genes in that main category. According to participation in KEGG metabolic pathways, genes can be divided into five branches: A, cellular processes; B, environmental information processing; C, genetic-based information processing; D, metabolism; E, organismal systems.

The GO classification demonstrated that 66,886 sequences could be assigned into 48 functional groups (Figure 1A). In the three main categories of the GO classification, “metabolic process,” “binding,” and “cell and cell part” terms were dominant, respectively.

The GO analysis showed that the functions of the identified genes involved various biological processes. Totally, 40,256 unigenes were annotated in the “metabolic process” category, 38,541 unigenes were annotated in the “cellular process” category, and 36,898 unigenes were annotated in the “binding” category (Figure 1A).

### KOG classification

In total, 45,832 unigenes were categorized into 26 functional groups (Figure 1B). The main groups were “post-translational modification, protein turnover, chaperone” (5,184 unigenes), “general functional prediction only” (7,232 unigenes), and “signal transduction” (4,453 unigenes). These results demonstrated that based on high-throughput sequencing, novel genes that might play roles in CryAc resistance can be identified (Figure 1B).

### Functional classification by KEGG

The KEGG classification revealed that 35,802 annotated unigenes were mapped to the reference canonical pathways in KEGG and categorized into 5 KEGG pathways. The unigenes were clustered into various classifications, including metabolism (25,537 members), organismal systems (13,554 members), genetic information processing (8,519 members), cellular processes (6,849 members), and environmental information processing (5,583 members). These annotations of unigenes provide a valuable resource for investigating functions, specific processes, and pathways in cotton bollworm Cry1Ac-resistance research (Figure 1C).

### Estimates of differential expression among the midgut transcripts

The DGE was used to analyze gene expression among the seven strains, including one susceptible and six Cry1Ac-resistant strains. Fourteen DGE libraries (containing two biological replicates): LF-1, LF-2, LF5-1, LF5-2, LF10-1, LF10-2, LF20-1, LF20-2, LF30-1, LF30-2, LF60-1, LF60-2, LF120-1, and LF120-2 were sequenced and between 6.4 and 11.3 million clean reads were generated. The number of clean read entities with unique nucleotide sequences ranged from 5,935,643 to 10,511,449 (Table 2). Moreover, 94.62% (9,885,875) of the sequences in the transcriptome database were unequivocally identified by unique genes (Table 2).

**Table 2:** Statistics of DGE sequencing.

**Sample name**	**Raw reads**	**Clean reads**	**Total mapped**
LF-1	8,255,755	8,148,765	7,608,627 (93.37%)
LF-2	8,682,852	8,582,319	8,034,691 (93.62%)
LF5-1	8,952,571	8,843,835	8,265,273 (93.46%)
LF5-2	9,185,832	9,074,638	8,531,159 (94.01%)
LF10-1	8,541,149	8,439,579	7,869,732 (93.25%)
LF10-2	8,955,009	8,832,499	8,226,818 (93.14%)
LF20-1	9,897,128	9,778,861	9,156,576 (93.64%)
LF20-2	8,953,526	8,848,022	8,361,765 (94.50%)
LF30-1	9,603,201	9,474,704	8,762,324 (92.48%)
LF30-2	11,407,539	11,269,610	10,511,449 (93.27%)
LF60-1	10,218,887	10,091,483	9,491,482 (94.05%)
LF60-2	10,582,957	10,448,394	9,885,875 (94.62%)
LF120-1	6,433,103	6,361,846	5,935,643 (93.30%)
LF120-2	10,907,563	10,792,334	10,015,635 (92.80%)

### Differentially expressed genes in different resistant developmental strains

**Figure 2:**
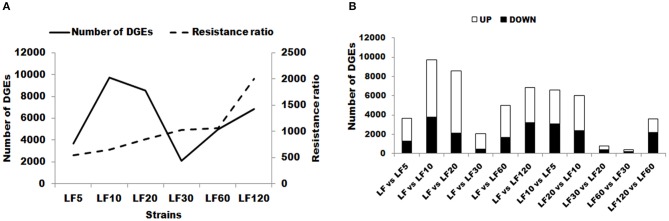
Changes in gene expression profiles among the different resistant strains. **(A)** The number of upregulated and downregulated genes between LF and LF5; LF and LF10; LF and LF20; LF and LF30; LF and LF60; LF and LF120; LF10 and LF5; LF20 and LF10; LF30 and LF20; LF60 and LF30; and LF120 and LF60 are summarized. Up: upregulated in LF-resistant strains; Down: downregulated in LF-resistant strains. **(B)** The number of total changes in gene expression in each resistant strain and resistance ratio of each resistant strain are summarized. DEGs, differentially expressed genes.

The DEG numbers detected to confer resistance level in six resistant LF strains did not increase along with the increase of resistance level to Cry1Ac (Table 1; Figure 2); the changes in the trends in the numbers of DEGs in six resistant LF strains are similar to the letter “N” in Figure 2A. Commonly, more upregulated genes than downregulated genes were detected in each of the six resistant LF strains (Figure 2A). Compared with the susceptible LF strain, 3,688 unigenes were expressed differentially in the LF5 strain, which presents the lowest resistance level. The highest numbers of DGEs occurred in the LF10 strain (9,712) followed by the LF20 stain (8,558) (Figure 2B). The lowest numbers of DGEs occurred in the LF30 strain (2,085) (Figure 2). Although LF60 showed the same resistance level as LF30, more DEGs were found in the LF60 strain (4,990), possibly due to the greater exposure to Cry1Ac toxin for the LF60 strain (Table 1; Figure 2). In total, 6,859 unigenes were expressed differentially in the LF120 strain, although the larvae of this strain showed the highest resistance. Comparing two neighboring strains, more genes showed significant differences in expression levels between LF10 vs. LF5 and between LF20 vs. LF10, and fewer genes showed significant differences in expression levels between LF30 vs. LF20 and between LF60 vs. LF30 (Figure 2A). However, the declining trend did not continue between LF120 vs. LF60, possibly due at least in part to the greater number of mutations in more genes or alleles involved in conferring a higher resistance level in the LF120 strain (Figure 2A). To analyze the function of DEGs between the LF and LF-resistant strains, these genes were classified in GO terms. The 30 significantly enriched (according to the corrected pValue) GO terms are shown in Figure S2. The differentially expressed genes showed significant enrichment in “catalytic activity,” “endopeptidase activity,” “aminopeptidase activity,” “serine-type endopeptidase activity,” “proteolysis,” “biological process,” “metabolic process,” “peptidase activity,” “serine-type peptidase activity,” “metallopeptidase activity,” “exopeptidase activity,” “hydrolase activity,” “serine hydrolase activity,” “protein metabolic process,” “peptidase activity,” “acting on L-amino acid peptides,” and “organic substance metabolic process” terms in all resistant strains (Table 3). These DEGs may help the cotton bollworm to enhance their physiology to adapt to the Cry1Ac toxin. The LF10, LF20, LF30, and LF60 have moderate resistance level and some of the differentially expressed genes in these four resistant strains were significantly enriched in “ribosome,” “translation,” “non-membrane-bounded organelle,” “ribonucleoprotein complex,” “structural constituent of ribosome,” and “intracellular non-membrane-bounded organelle” (Table 3). The LF5 has the least resistance and some of the differentially expressed genes had the functions related to xenobiotics because they showed significant enrichment in “xenobiotic metabolic process,” “response to xenobiotic stimulus,” “antioxidant activity,” “cis-stilbene-oxide hydrolase activity,” “coenzyme binding,” “cellular response to chemical stimulus,” and “cellular response to xenobiotic stimulus” (Table 3; Figure S2). As the resistance level increased, the genes of the cotton bollworms showed some significant differences in macromolecule metabolic processes in the LF10 and LF20 strains (Table 3; Figure S3). Different from other strains, some genes involved in “proton-transporting V-type ATPase complex” and “proton-transporting two-sector ATPase complex” showed significant changes in the LF30 strain (Table 3; Figure S2). For the LF60 strain, some genes involved in “chitin binding,” “sterol binding,” and “alcohol binding” showed more significant expression differences than other Go terms, and this was specifically true in this strain (Table 3; Figure S2). As the highest resistance-level strain LF120, the differentially expressed genes were enriched more significantly in “cellular iron ion homeostasis,” “ferric iron binding,” “hexachlorocyclohexane metabolic process,” “chlorinated hydrocarbon metabolic process,” “halogenated hydrocarbon metabolic process,” “cellular transition metal ion homeostasis,” “transition metal ion homeostasis,” and “iron ion homeostasis” (Table 3; Figure S2). The enrichment of DEGs in the same or in different pathways provides information that can aid in understanding the development of resistance and the resistance mechanisms in different strains.

**Table 3:** Gene ontology (GO) classification of differentially expressed genes in susceptible and resistant strains.

**GO_accession**	**Description**	**LF vs. LF5**	**LF vs. LF10**	**LF vs. LF20**	**LF vs. LF30**	**LF vs. LF60**	**LF vs. LF120**
GO:0003824	Catalytic activity	1,770	4,306	3,729	1,002	2,388	3,133
GO:0004175	Endopeptidase activity	317	769	642	246	464	566
GO:0004177	Aminopeptidase activity	49	130	109	54	95	98
GO:0004252	Serine-type endopeptidase activity	207	527	407	160	300	372
GO:0006508	Proteolysis	410	1,008	793	284	554	727
GO:0008150	Biological_process	2,366	6,006	5,205	1,374	3,279	4,258
GO:0008152	Metabolic process	2,020	4,970	4,314	1,164	2,747	3,528
GO:0008233	Peptidase activity	425	1,036	831	296	583	756
GO:0008236	Serine-type peptidase activity	237	614	467	180	341	417
GO:0008237	Metallopeptidase activity	131	255	208	88	167	212
GO:0008238	Exopeptidase activity	104	238	173	75	141	185
GO:0016787	Hydrolase activity	912	2218	1,950	573	1,214	1,618
GO:0017171	Serine hydrolase activity	237	614	467	180	341	417
GO:0019538	Protein metabolic process	827	2,087	1,815	562	1,181	1,484
GO:0070011	Peptidase activity, acting on L-amino acid peptides	414	993	796	292	566	730
GO:0071704	Organic substance metabolic process	1672	4,198	3,667	968	2,295	2,911
GO:0005840	Ribosome		560	483	157	321	
GO:0006412	Translation		612	572	178	358	
GO:0030529	Ribonucleoprotein complex		621	541	171	356	
GO:0043228	Non-membrane-bounded organelle		936	835	245	510	
GO:0043232	Intracellular non-membrane-bounded organelle		936	835	245	510	
GO:0003735	Structural constituent of ribosome		404	349	125	231	
GO:0006805	Xenobiotic metabolic process	36					
GO:0009410	Response to xenobiotic stimulus	36					
GO:0016209	Antioxidant activity	51					
GO:0033961	Cis-stilbene-oxide hydrolase activity	12					
GO:0050662	Coenzyme binding	153					
GO:0070887	Cellular response to chemical stimulus	49					
GO:0071466	Cellular response to xenobiotic stimulus	36					
GO:0043170	Macromolecule metabolic process		3,006	2,686			
GO:0016469	Proton-transporting two-sector ATPase complex				81		
GO:0033176	Proton-transporting V-type ATPase complex				45		
GO:0008061	Chitin binding					70	
GO:0032934	Sterol binding					25	
GO:0043178	Alcohol binding					25	
GO:0006879	Cellular iron ion homeostasis						38
GO:0008199	Ferric iron binding						38
GO:0019497	Hexachlorocyclohexane metabolic process						36
GO:0042196	Chlorinated hydrocarbon metabolic process						36
GO:0042197	Halogenated hydrocarbon metabolic process						36
GO:0046916	Cellular transition metal ion homeostasis						38
GO:0055072	Iron ion homeostasis						38
GO:0055076	Transition metal ion homeostasis						38

The most significantly enriched pathways are shown between susceptible and resistant strains.

In organisms including cotton bollworm, different genes possess special biological functions and coordinate with each other. In another way, through KEGG pathway analysis, significant enrichment can identify DGEs that are involved in the main biochemical pathways and signal transduction pathways. The most significantly enriched (according to the corrected *p*-value) 20 KEGG pathways are shown in Figure S3. The results indicated that the DEGs were enriched more significantly in “propanoate metabolism,” “two-component system,” and “protein processing” in the endoplasmic reticulum in all resistant strains (Table 4). In the five least-resistant strains, the differentially expressed genes were enriched more significantly in “citrate cycle (TCA cycle)” and “carbon fixation pathways in prokaryotes.” For the LF5 strain, some DEGs were enriched significantly in “starch and sucrose metabolism” and in “plant-pathogen interaction” pathways. For the LF10 strain, some differentially expressed genes were enriched significantly in “ABC transporters” pathways (Table 4) and other genes that differentially expressed between from LF5 and LF 10 were involved significantly in “glutathione metabolism,” a pathway that may help to detoxify Cry1Ac toxins. Some DEGs from LF30 were found to be involved significantly in the “mTOR signaling pathway” (Table 4), which is a crucial signaling pathway that mediated cell growth and proliferation (Kazuyoshi Yonezawaa, 2004). These results indicated that these genes in LF30 may affect larval growth. Further details of differentially expressed genes that were enriched significantly in the KEGG pathway are shown in Table 4 and Figure S3.

**Table 4:** The KEGG ortholog classification of differentially expressed genes between the susceptible and the resistant strains.

**#Term**	**Description**	**LF vs. LF5**	**LF vs. LF10**	**LF vs. LF20**	**LF vs. LF30**	**LF vs. LF60**	**LF vs. LF120**
ko00500	Starch and sucrose metabolism	65					
ko04626	Plant-pathogen interaction	22					
ko00480	Glutathione metabolism	63	119				
ko01120	Microbial metabolism in diverse environments	182	317	269			
ko00020	Citrate cycle (TCA cycle)	68	119	98	29	72	
ko00720	Carbon fixation pathways in prokaryotes	22	49	42	14	28	
ko00640	Propanoate metabolism	45	77	61	19	47	64
ko02020	Two-component system	27	49	35	24	37	32
ko04141	Protein processing in endoplasmic reticulum	91	186	164	46	120	161
ko00627	Aminobenzoate degradation		41	30	16	27	
ko00630	Glyoxylate and dicarboxylate metabolism		54	47	19	42	
ko04721	Synaptic vesicle cycle		81	79	43		
ko04966	Collecting duct acid secretion		63	63	35		
ko04975	Fat digestion and absorption		42	35	15		
ko02010	ABC transporters		51				
ko04380	Osteoclast differentiation		33				
ko00362	Benzoate degradation			33			
ko00564	Glycerophospholipid metabolism			57			
ko00710	Carbon fixation in photosynthetic organisms			48			
ko00970	Aminoacyl-tRNA biosynthesis			80			
ko03060	Protein export			37			
ko03010	Ribosome				115		
ko04150	mTOR signaling pathway				14		
ko04210	Apoptosis				7		
ko04530	Tight junction				26		
ko00281	Geraniol degradation					12	
ko00565	Ether lipid metabolism					17	
ko04146	Peroxisome					80	
ko00260	Glycine, serine and threonine metabolism						70
ko04614	Renin-angiotensin system						27

*The most significantly enriched pathways are shown between susceptible and resistant strains*.

### Expression level of trypsin genes involved in development of resistance

**Figure 3:**
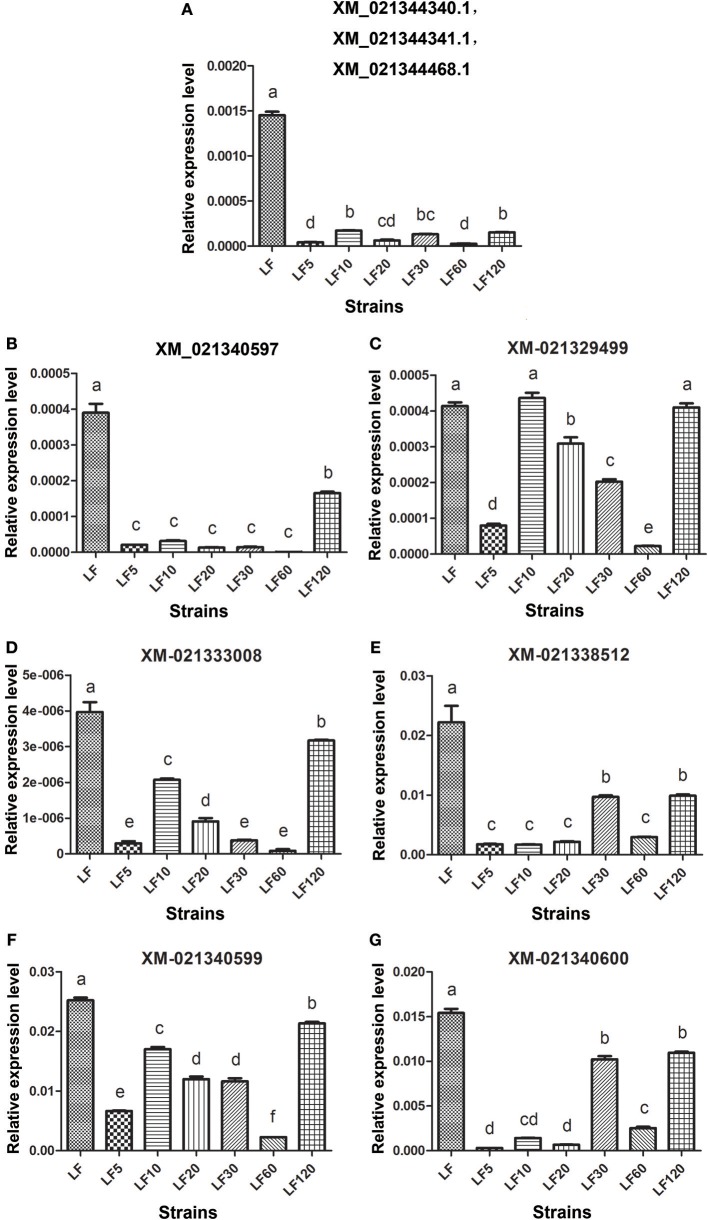
The qRT-PCR analysis of transcript abundances among candidate Bt resistance trypsin genes within *H. armigera*-susceptible (LF) and -resistant (LF5, LF10, LF20, LF30, LF60, and LF120) strains. **(A)** The three genes ID numbers represent the three trypsin genes in NCBI; a pair of primers in the conserved region of these three trypsin genes were used to analyze transcript abundances. **(B-G)** Represent the relative expression levels of trypsin genes of XM_021340597, XM_021329499, XM_021333008, XM_021338512, XM_021340599 and XM_021340600 in resistance strains, respectively. Values shown are means and standard errors. Different letters indicate significant differences between treatments (*P* < 0.05; HSD test).

The trypsin family is present widespread in animals and plays a variety of roles, especially in the digestive system. Lower expression and activity of trypsin proteases result in decreased activation of the Cry1Ac protoxin and is a mechanism of resistance to Cry1Ac in *H. armigera* (Liu et al., 2014; Wei et al., 2016a). Fourteen unigenes (comp35781_c0_seq1, my_s32485, comp41058_c1_seq5, Unigene12105, Unigene47996, Unigene20462, Unigene36451, Unigene15742, Unigene43312, Unigene8736, comp41058_c4_seq1, Unigene31995, my_rep_c15473, and my_rep_c25150) were matched to nine corresponding candidate trypsin genes (Gene bank: XM_021340600, XM_021340599, XM_021340597, XM_021333008, XM_021329499, XM_021338512, XM_021344340.1, XM_021344341.1, and XM_021344468.1), and the mRNA levels of these genes were found to be decreased significantly in resistant strains in comparison with the susceptible LF strain (Table 5), consistent with the qRT-PCR results obtained in all six resistant strains (Figure 3).

**Table 5:** Estimates of transcripts levels (expression) among candidate Cry1Ac toxin-resistance genes in *H. armigera* midgut based on DGE sequence mapping to each unigene.

**Gene_id**	**Annotation**	**LF vs. LF5**	**LF vs. LF10**	**LF vs. LF20**	**LF vs. LF30**	**LF vs. LF60**	**LF vs. LF120**	**Gene bank:**
comp35781_c0_seq1	Trypsin	+1.74 (0.03769)	+6.10 (2.69E-24)	NS	NS	+3.02 (0.00053226)	+2.38 (0.0003760)	XM_021340600
my_s32485	Trypsin	NS	Inf (7.87E-47)	Inf (8.57E-28)	NS	+3.41 (7.50E-05)	Inf (3.67E-38)	XM_021340600
comp41058_c1_seq5	Trypsin	Inf (4.39E-06)	+4.51 (0.0001879)	NS	NS	NS	+4.68 (0.0004067)	XM_021340599
Unigene12105	Trypsin	3.74 (0.018208)	3.13 (0.024922)	NS	NS	NS	5.34 (0.00027642)	XM_021340597
Unigene47996	Trypsin	4.02 (0.010678)	4.35 (0.0021366)	NS	NS	NS	6.81 (6.72E-05)	XM_021340597
Unigene20462	Trypsin	2.69 (0.0017737)	3.72 (4.98E-05)	2.44 (0.018081)	5.38 (0.019653)	2.94 (0.0012445)	NS	XM_021333008
Unigene36451	Trypsin	3.02 (0.021797)	Inf (3.54E-09)	8.01 (1.32E-07)	NS	Inf (5.07E-09)	NS	XM_021333008
Unigene15742	Trypsin	2.31 (0.002246)	NS	2.86 (0.0003248)	NS	4.73 (1.12E-07)	NS	XM_021329499
Unigene43312	Trypsin	1.93 (0.014007)	1.77 (0.034651)	NS	NS	NS	2.52 (1.72E-05)	XM_021338512
Unigene8736	Trypsin	Inf (1.71E-25)	11.19 (4.12E-32)	Inf (4.17E-21)	Inf (8.40E-06)	Inf (1.25E-19)	10.84 (5.25E-27)	XM_021338512
comp41058_c4_seq1	Trypsin	2.41 (0.0024489)	3.39 (4.54E-06)	NS	NS	NS	7.49 (7.84E-15)	XM_021344340.1
Unigene31995	Trypsin	2.34 (0.0030509)	3.12 (5.28E-06)	NS	NS	3.12 (0.00049004)	6.79 (1.80E-14)	XM_021344341.1
my_rep_c15473	Trypsin	2.65 (0.0086791)	4.50 (6.56E-06)	NS	NS	2.42 (0.013659)	9.08 (7.29E-13)	XM_021344468.1
my_rep_c25150	Trypsin	2.13 (0.018342)	2.96 (7.41E-05)	NS	NS	2.84 (0.002339)	7.22 (1.28E-12)	XM_021344468.1
Unigene39578	ALP-like	−8.57 (2.87E-12)	−12.34 (6.09E-28)	−7.61 (1.38E-07)	−9.45(0.00049)	−9.54 (7.26E-13)	−9.02 (6.24E-16)	XM_004928089.1
Unigene40436	ALP-like	−6.23 (0.00281)	−6.47 (0.000213)	NS	NS	−6.09 (0.012926)	−6.45 (0.000193)	XM_004928089.1
comp36273_c0_seq1	ALP-like	−10.29 (0.047997)	−9.50 (2.26E-32)	−8.03 (1.54E-14)	−8.56 (0.03282)	−11.00 (0.000246)	−5.99 (4.16E-09)	XM_004928089.1
Unigene29103	ALP-like	# (0.033832)	# (6.87E-11)	NS	NS	# (0.00015902)	# (0.0011264)	XM_004928089.1
Unigene5645	ALP-like	NS	# (0.0025752)	# (0.00017963)	# (0.020973)	# (1.45E-11)	NS	XM_004928089.1
Unigene6346	ALP-like	# (1.81E-16)	# (5.77E-26)	# (4.11E-13)	# (4.67E-05)	# (1.21E-17)	# (1.03E-24)	XM_004928089.1
Unigene6678	ALP-like	# (0.029709)	# (0.047379)	# (0.041528)	# (0.002726)	# (3.54E-05)	NS	XM_004928089.1
Unigene29103	ALP-like	# (0.033832)	# (6.87E-11)	NS	NS	# (0.000159)	# (0.001126)	XM_004928089.1
Unigene33242	ALP-like	# (7.14E-05)	# (1.44E-07)	# (5.80E-05)	# (0.010562)	# (7.85E-09)	# (3.98E-07)	XM_004928089.1
Unigene37010	ALP-like	# (2.78E-05)	# (5.02E-06)	# (0.001702)	# (0.013456)	# (1.07E-07)	# (4.58E-06)	XM_004928089.1
Unigene40165	ALP-like	NS	# (5.79E-12)	# (0.036779)	NS	# (7.72E-07)	NS	XM_004928089.1
Unigene40714	ALP-like	# (1.42E-05)	# (1.14E-10)	# (0.005822)	# (0.002332)	# (3.43E-09)	# (1.96E-12)	XM_004928089.1
Unigene40726	ALP-like	# (3.83E-10)	# (5.63E-15)	# (4.72E-05)	# (0.001478)	# (1.14E-09)	# (3.54E-12)	XM_004928089.1
Unigene49748	ALP-like	# (3.81E-07)	# (4.16E-12)	# (0.004921)	# (0.015371)	# (3.70E-07)	# (1.68E-09)	XM_004928089.1
comp33523_c0_seq1	ALP2	+3.27 (0.000188)	+2.76 (0.000158)	+3.51 (0.000204)	NS	NS	NS	EU729323.1
Contig1878	APN5	−3.22 (2.20E-05)	−2.13 (0.0030248)	NS	NS	−3.52 (0.0001526)	−0.306 (1.43E-05)	AY894814.1
Unigene13560	APN5	−6.25 (0.002507)	−7.72 (2.85E-09)	−8.74 (4.40E-07)	NS	−8.57 (2.59E-13)	−8.64 (3.44E-09)	EU325551.1
Unigene13606	APN5	NS	−5.84 (0.004192)	−6.62 (0.0001361)	NS	−6.25 (0.0041708)	−6.25 (0.0005066)	EU325551.1
Unigene39423	APN5	NS	−7.49 (5.38E-05)	−8.43 (4.42E-10)	NS	−7.95 (3.36E-07)	−7.73 (6.47E-09)	EF417486.1
Unigene5729	APN5	−6.62 (0.0002943)	−8.01 (8.60E-11)	−8.54 (9.51E-08)	−7.03 (0.04486)	−8.58 (3.15E-09)	−8.31 (1.21E-11)	EF417486.1

*The transcripts are significantly up-(-) or downregulated (+) in six Cry1Ac selected lines (LF5, LF10, LF20, LF30, LF60, and LF120) and are based on log_2_(fold-change) estimates compared to common susceptible strain (LF). NS, not significant; -Inf, not detected expression in LF-susceptible strain; #, not detected expression in Cry1Ac-resistant strains. Corrected P-value of 0.05 and the absolute value of log_2_(fold-change) of 1 were set as the threshold for significantly differential expression*.

### Expression level of Cry1Ac-receptors genes involved in development of resistance

**Figure 4:**
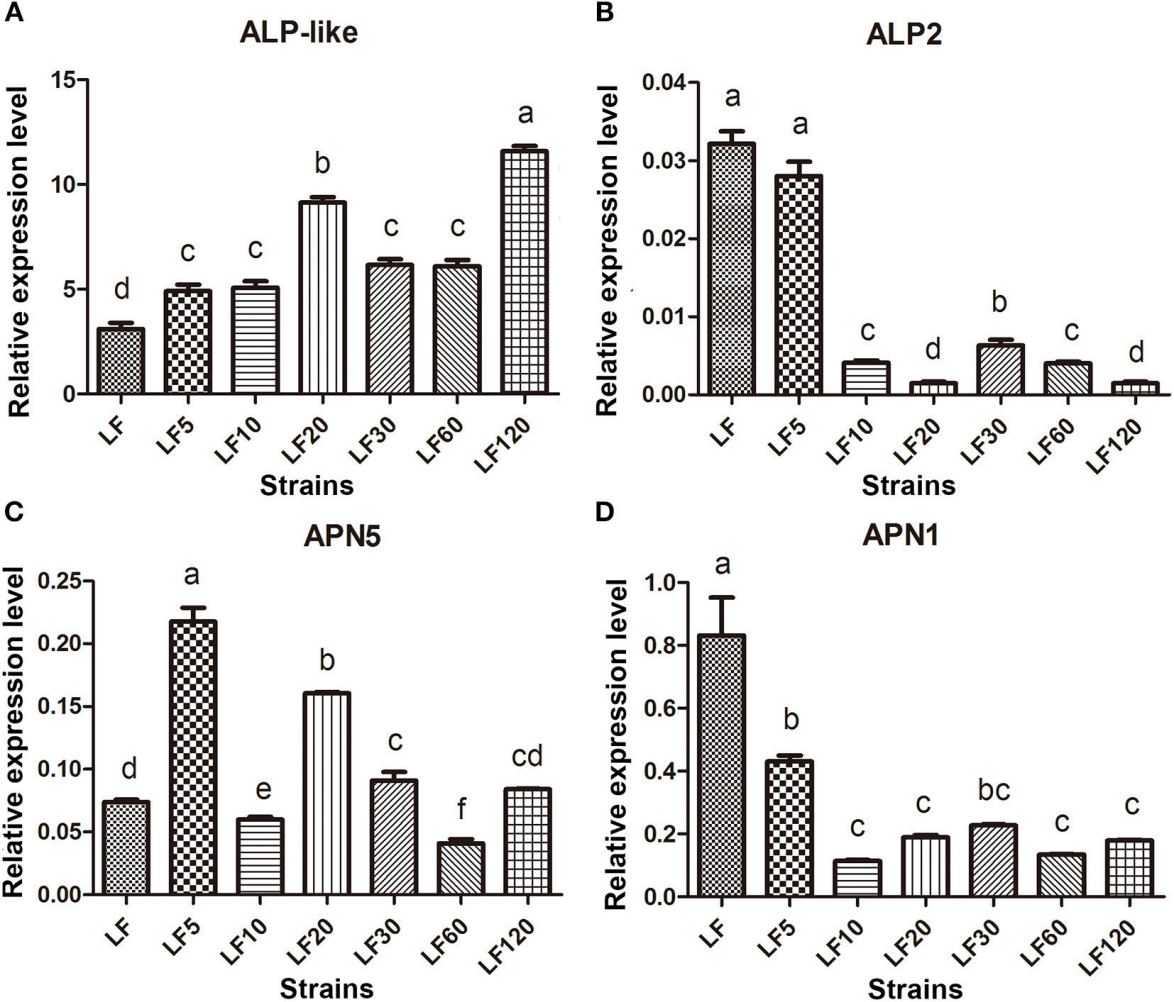
The qRT-PCR estimates of transcript abundances among candidate Bt-resistance receptor genes within *H. armigera*-susceptible (LF) and -resistant (LF5, LF10, LF20, LF30, LF60, and LF120) strains. **(A-D)** Represent the relative expression levels of ALP-like, ALP2, APN5 and APN1 in resistance strains, respectively. Values shown are means and standard errors. Different letters indicate significant differences between treatments (*P* < 0.05; HSD test).

Several known Bt receptors and Bt-resistance genes including ALP-like (XM_004928089.1), ALP2 (EU729323.1), and APN5 (AY894814.1, EU325551.1, and EF417486.1) showed the same changed trend in all resistance strains (Table 5). The ALP-like genes encoded by fourteen unigenes (Unigene39578, Unigene40436, comp36273_c0_seq1, Unigene29103, Unigene5645, Unigene6346, Unigene6678, Unigene29103, Unigene33242, Unigene37010, Unigene40165, Unigene40714, Unigene40726, and Unigene49748) were found to be upregulated significantly based on DGE results (Table 5) and these results were further verified by qRT-PCR in all six resistant strains (Figure 4A). Another ALP gene, ALP2 (comp33523_c0_seq1), was significantly upregulated in the LF5, LF10, and LF20 strains, but there was no significant change in LF30, LF60, and LF120. However, qRT-PCR analysis demonstrated that this ALP2 (comp33523_c0_seq1) of *H. armigera* was ubiquitous and significantly reduced in all six resistant strains (Figure 4B). The APN5 (Contig1878, Unigene13560, Unigene13606, Unigene39423, and Unigene5729) was upregulated significantly in the LF-resistant strains according to DGE results (Table 3), However, qRT-PCR analysis indicated that this gene was upregulated significantly in the LF5, LF20, and LF30 strains, unchanged in the LF120 strain, and significantly downregulated in the LF10 and LF60 strains (Figure 4C). In contrast, APN1 (AF441377) was downregulated significantly in all these six resistance strains according to the qRT-PCR analysis, but not according to the DGE results (Figure 4D).

## Discussion

Resistance to Cry1Ac is controlled by multiple genes involved in fitness costs and in the selection of recessive or dominant receptors (and even alleles with different types of mutations on the same locus) and their interactions (Tabashnik et al., 2005; Xu et al., 2005; Zhang et al., 2009, 2012; Gahan et al., 2010; Baxter et al., 2011; Jurat-Fuentes et al., 2011; Atsumi et al., 2012; Xiao et al., 2014; Chen et al., 2015). Fitness costs contain longer period of development and reduction in survival, pupal weight, and fecundity (Sayyed et al., 2008). Fitness costs are expected to increase steadily with the development of increased resistance (Cao et al., 2014). According to Cao et al. (2014), who established multiple regressions to predict overall fitness cost and resistance level with fitness costs, these LF-resistant strains may use a second phase of resistance. In this stage, resistance gene-encoding enzymes, such as digestive enzymes, hydrolase, detoxification enzymes, and catalytic enzymes are considered the most important factor to produce fitness cost (Rajagopal et al., 2009; Zhu et al., 2011; Guo et al., 2012; Cao et al., 2013; Lei et al., 2014; Liu et al., 2014; Wei et al., 2016a; Zhang et al., 2017). As a significant and universal phenomenon, we found in this study that a significant portion of DEGs were enriched predominantly in “catalytic activity,” “endopeptidase activity,” “aminopeptidase activity,” “serine-type endopeptidase activity,” “proteolysis,” “biological process,” “metabolic process,” “peptidase activity,” “metallopeptidase activity,” “serine-type peptidase activity,” “exopeptidase activity,” “serine hydrolase activity,” “hydrolase activity,” “protein metabolic process,” “acting on L-amino acid peptides,” “peptidase activity,” and “organic substance metabolic process” for all the resistant strains (Table 3; Figure S2). High-level expression of the genes involved in the earlier-mentioned pathways will help resistant insects to avoid Cry1Ac damage. However, in compensation, these resistant insects develop a lower hatching rate, a lower copulation rate, a lower emergence rate, and even a lower survival rate (Cao et al., 2014). These findings suggest that the resistance to Cry1Ac in *H. armigera* might also be associated with increased catalytic activity, digestive activity, hydrolase activity, and detoxification activity.

The current understanding of Bt-toxin resistance in insects is associated generally with either conversion of Bt protoxins to activated toxins by insect midgut proteases (Rajagopal et al., 2009; Cao et al., 2013; Liu et al., 2014; Wei et al., 2016a) or by altered binding capacity of toxins to midgut proteins (Xu et al., 2005; Zhang et al., 2009, 2012; Gahan et al., 2010; Baxter et al., 2011; Jurat-Fuentes et al., 2011; Atsumi et al., 2012; Xiao et al., 2014; Chen et al., 2015). Decreased transcript levels of trypsins have been associated with reduced Cry1Ac protoxin activation (Rajagopal et al., 2009; Liu et al., 2014). This result is consistent with our present results, in which nine transcripts encoding trypsin serine protease were found to be downregulated in six LF-resistant strains (Table 5; Figure 3). Previous studies also found that reduced trypsinlike activity is correlated with reduced expression levels of trypsin gene transcripts in the LF120 strain (Wei et al., 2016a). Similar results were also reported in *Ostrinia nubilalis*, in which defense against Bt toxins was considered as main mechanism of resistance (Yao et al., 2012; Nanoth Vellichirammal et al., 2015). These results revealed that reduced protoxin activation is considered generally as a resistance mechanism against Bt proteins. More importantly, for the first time, we revealed that these nine trypsin serine proteases downregulated in Cry1Ac-resistant *H. armigera* was probably a common phenomenon. This result indicated that some trypsin activators may be used to improve the toxicity of Bt to a certain degree.

High levels of resistance are most commonly associated with mutations that disrupt the binding of Cry proteins to midgut receptors, decreased expression of these specific receptors in the midgut, and decreases of the toxin binding to its midgut proteins in the resistant strain (Ferré and Van Rie, 2002; Wu, 2014). While investigating the universal mechanism of resistance to Bt proteins, downregulation of ALP2 and APN1 and upregulation of ALP-like were found in these LF-resistant strains (Table 5; Figure 4). Reduced activity and transcription of ALP, which binds Cry1Ac, caused the resistance to Cry1Ac in the LF10, LF30, LF60, and LF120 strains when compared with a 96S-susceptible strain (Chen et al., 2015). The ALP1 and ALP2 all had been reported as the receptors of *H. armigera* (Ning et al., 2010; Chen et al., 2015); however, the total ALP activity was decreased and the transcription of the conserved region of HaALP2 (accession no. EU729323) and HaALP1 (accession no. EU729322) isoforms was also reduced in the resistance strains. It was difficult to know whether ALP1 or ALP2 caused the resistance to Cry1Ac. Here, we pinpoint accurately that the downregulation of ALP2 transcription leads to Cry1Ac resistance in LF resistance. Moreover, in LF Cry1Ac-resistant *H. armigera* larvae, a decrease in ALP activity may be correlated with reduced levels of ALP2 transcripts. However, ALP1 seems not to be a gene that importantly associated with resistance to Bt Cry1Ac since ALP1 was verified to be upregulated in all the LF-resistant strains (Zhang, 2013, PhD thesis).

In our study, ALP1 and ALP-like (Table 5; Figure 4) were found to be upregulated in all these LF-resistant strains. The increased expression of both genes in the LF-resistant strains was associated probably with the gut defensive response to Cry1Ac intoxication. In fact, ALP expression is considered as a marker for stem cell proliferation, which is crucial to gut defensive responses to Cry toxins (Singh et al., 2012). Moreover, midgut regeneration has been proposed as a mechanism of Cry1Ac resistance in *H. virescens* (Forcada et al., 1999). Further studies are needed to determine the molecular mechanisms responsible for the upregulation of ALP1 and ALP-like in these LF-resistant larvae.

Various studies with glycosylphophatidylinositol (GPI)-anchored APN1 from lepidopteran insects consistently demonstrated that APN1 is one of the midgut receptors for Cry1Ac and related to the resistance to Bt toxins (Zhang et al., 2009; Tiewsiri and Wang, 2011; Valaitis, 2011; Flores-Escobar et al., 2013; Wei et al., 2016b). However, our DGE data analysis demonstrated that the expression level of APN1 transcript was not significantly different between the LF-susceptible and -resistant strains. This result contradicted our qRT-PCR analysis (Figure 4), which demonstrated that APN1 transcript was decreased significantly in the LF-resistant strains (Figure 4). The discrepancy between the DGE analysis and the qRT-PCR analysis may be due to the sensitivity of qRT-PCR, which is higher than that for DEG. The APN5 can also bind to Cry1Ac in *H. armigera* (Wang et al., 2005), but its involvement in Cry1Ac resistance has not been documented. In this study, we found that APN5 is widely upregulated in the LF-resistant strains using DGE analysis, and this result was confirmed further by qRT-PCR in the LF5, LF20, LF30, and LF120 strains. The upregulation of APN6 has been reported to act as a compensation of APN1 loss in order to minimize the fitness costs of resistance in *Trichoplusia ni* (Tiewsiri and Wang, 2011). Whether a similar function of APN5 exists in LF5, LF20, LF30, and LF120 *H. armigera* warrants further study. Other Cry1Ac receptors (cadherin and ABC transports) and related genes showed different expression levels in individual LF-resistant strains, but they did not show a universal mechanism of resistance to Cry1Ac in all LF-resistant strains. This finding suggests that changes in expression of one or more of the Cry1Ac receptors and related genes can influence Cry toxin resistance traits to different degrees. Indeed, additional study is required to decipher the individual roles of the interactions between Bt receptors within the framework of toxin modes of actions. Nevertheless, our results provide evidence that the downregulation of ALP2 and APN1 affected the resistance in both lower and higher levels of resistance. Therefore, these genes may be used as markers to monitor and manage pest resistance in transgenic crops.

Lei et al. (2014) identified unigenes that are differentially expressed between Cry1Ac-susceptible and two resistant *Plutella xylostella* strains by RNA-seq analysis, and further analysis found that the higher resistance strain showed the greater number of EDUs. However, the higher resistance to Cry1Ac in insects does not always involve the use of more DEGs to adapt to more toxins. Our results showed the numbers of DEGs increased from LF5 to LF10 and LF30, LF60 to LF120, consistent with the resistance ratios of these strains (Figure 2B). However, the DEG numbers in the LF30, LF60, and LF120 strains were all lower than those in the LF10 and LF20 and a negative correlation was found between DEG numbers and resistance ratios in the LF10, LF20, and LF30 strains (Figure 2). Obviously, our data indicate that this is in response to different selection pressures. Different genes are used to adapt to the new environment, including the evolution of resistance to Cry1Ac, finally developing different resistant mechanisms. This conclusion was confirmed further by previous studies in these LF-resistant strains. For example, the cis-mediated downregulation of HaTryR expression is considered as the main resistance mechanism in the LF5 strain (Liu et al., 2014). In the LF60 strain, an ABCC2 mutant (in which a 6bp deletion in genomic DNA introduces a premature stop codon) leads to the resistance to Cry1Ac (Xiao et al., 2014). Decreased ALP activity and transcription are considered to cause the Cry1Ac resistance in LF10, LF30, LF60, and LF120 (Chen et al., 2015). Although trypsin activity was decreased significantly in LF120, the high level of resistance to protoxin and activated toxin indicated that reduced activation of the protoxin was not a major Cry1Ac-resistance mechanism in LF120 (Wei et al., 2016a). Interestingly, the finding of the lowest number of DGEs indicated that a new domain mechanism of resistance has evolved in the LF30 strain (Figure 2). This result indicated that different genes and pathways were involved in Cry1Ac resistance in *H. armigera*. Also, these pathways seem to be differently affected depending on the level of resistance.

The GO and KEGG category analyses provided an important cue to uncover the different mechanisms involved in the development of resistance. First, the initial resistance appeared to increase immune and detoxification processes as shown by the series of DEGs in LF5 strains, predominantly DEGs involved in “xenobiotic metabolic process,” “response to xenobiotic stimulus,” “antioxidant activity,” “cis-stilbene-oxide hydrolase activity,” “coenzyme binding,” “cellular response to xenobiotic stimulus,” and “cellular response to chemical stimulus” (Table 3). These DEGs in the LF5 strain lead to increased immune and detoxification functions in the body, thus initiating defense to Bt invasion. Similar results were found in the KEGG pathway analysis, in which a series of DEGs were also enriched significantly in “plant-pathogen interaction,” “glutathione metabolism,” and “microbial metabolism in diverse environments” (Table 4). The changes of these DEGs in the LF5 stain indicated that low-level resistance is probably associated with insect immune and detoxification processes. With the increase of selection pressure involving Cry1Ac, the LF10 and LF20 strains showed different gene changes to that in the LF5 strain. These genes were found to participate in macromolecule metabolic process (Table 3) involved in the degradation, metabolism, transport, secretion, and absorption of macromolecular substances (Table 4; Figure S2). In particular, the 51 unigenes that encode ABC transporters were found to be expressed significantly in the LF10 strain. The ABC transporters, such as ABCG_1_, ABCC_2_, and ABCC_3_ have been confirmed to be Bt receptors and are related to the resistance to Bt (Xiao et al., 2014; Guo et al., 2015a,b; Tanaka et al., 2017). These results indicate that these genes are associated probably with insect resistance to Bt in the LF10 and LF20 strains. Convincing evidence shows that ABC transporters have been verified to be involved in the mode of Cry1Ac action and the mechanism of resistance to Cry1Ac.

The DEGs involved in “proton-transporting V-type ATPase complex” and “proton-transporting two-sector ATPase complex” were found to be expressed significantly in the LF30 strain (Table 3). It is not surprising that V-ATPase takes part in the resistance to Bt because previous reports have demonstrated that a number of V-ATPase subunits can bind to different Bt proteins, including Cry1Ab, Cry1Ac, and Cry4Ba (Bayyareddy et al., 2009; Chen et al., 2010; Xu et al., 2013). Similar results were reported in a study of a Cry1F-resistant strain, in which seven transcripts encoding V-ATPase subunits were identified significantly in downregulation (Nanoth Vellichirammal et al., 2015). It has been reported that V-ATPase subunits are involved in maintaining the alkaline conditions of the midgut (Onken et al., 2008). Further work should be carried out to verify the effect of V-ATPase subunits on midgut pH in the LF30 strain, as the results may provide further insight into resistance mechanisms used by this resistant strain.

At high levels of resistance to Cry1Ac, receptor mutations may be the main reasons underlying the resistance. However, the resistance mechanisms in LF60 have been identified (Xiao et al., 2014). In addition, other factors must be involved in the resistance to Cry1Ac because our results identified 4,990 DEGs that were expressed significantly in the LF60 strain, compared with the LF-susceptible stain. Interestingly, some DGEs were found to act in “chitin binding,” “sterol binding,” and “alcohol binding” (Table 3). Other DGEs were found to function in “geraniol degradation” and “ether lipid metabolism” based on the KEGG ortholog classification. Future studies are needed to determine the roles of these DGEs in the development of resistance in the LF60 strain.

Our previous study confirmed that trypsin activity was decreased significantly in LF120 (Wei et al., 2016a). Correspondingly, in the present study, nine trypsins were found to be significantly downregulated in the LF120 strain (Table 3). However, LF120 has the highest levels of mRNA for different trypsins among all resistant strains. These results indicate that the reduced activation of protoxin seems not to be a main mechanism of resistance to Bt proteins in this strain. Meanwhile, DGEs in LF120 were enriched significantly in “cellular iron ion homeostasis,” “ferric iron binding,” “cellular transition metal ion homeostasis,” “transition metal ion homeostasis,” and “iron ion homeostasis” (Table 3). This result suggested to us that ion homeostasis in the insect's body may play a important role in affecting the resistance to Bt, because unbalanced ion homeostasis can impair the normal functions of proteins within cells, hinder pore formation, and lead to cell death. As reported, Cry toxins can induce the formation of non-selective channels and then lead to imbalance of cations, anions, neutral solutes, and water, finally causing cell swelling and lysis (Knowles and Ellar, 1987). The role of ions, especially iron, in the mechanisms of resistance in the LF120 strain will be explored in future studies.

From LF5 strain to LF120 strain, many pathways seemingly exist. However, a domain pathway contributes hugely to Cry1Ac resistance. The upregulated or downregulated genes may not be fully illustrated in the resistance mechanisms that occur in the LF-resistant strains. Importantly, key genes in different pathways regulating the expressions of resistance-associated genes should be identified further. Also, these key genes may be used to modify *via* gene edition (CRISPR) for molecular control of the resistance. For example, the interplay between ALP and ABCC is controlled by MAP4K4 in the MAPK signaling pathway in *P. xylostella* (Guo et al., 2015b). However, as the firstly discovered pathways, V-ATPase, ABC transporters, or ion homeostasis, which are involved in Cry1Ac resistance, are lesser known, and more functional experiments need to be carried out in the future.

## Conclusion

Based on our observations, several factors are associated with Cry1Ac resistance in the LF-resistant *H. armigera* strains. Changes in catalytic activity, digestive activity, hydrolase activity, and detoxification activity and in the downregulations of receptors and related genes, including ALP2, APN1, and trypsin, unavoidably result in resistance to Cry1Ac. The ALP2 and APN1 can, therefore, be considered as probes to monitor the resistance of *H. armigera* to first-generational Cry1Ac crops in the field. Most importantly, the results here revealed multiple genes and pathways that are probably involved in resistance. Also, these pathways seem to be differently affected depending on the level of resistance. For controlling the lower level Cry1Ac-resistance, some enzyme inhibitors or activators can be explored to improve the toxicity of Bt. As the resistance increases, the catalytic activity, digestive activity, hydrolase activity, and detoxification activity may not be the main role of resistance. Special pathways and genes may be involved in the resistance, such as V-ATPase, ABC transporters, or ion homeostasis, and they seem to differently contribute to resistance depending on the level of resistance. The identification of the key genes that regulate the main pathway contributing to resistance are underway. Also, these genes could be used *via* gene edition (CRISPR) for molecular control of resistance.

## Data accessibility statement

Original sequencing reads are available from the GenBank Under accession numbers: PRJNA451313.

## Author contributions

JW and GL conceived and designed the experiments. JW, LC, and SY performed the experiments. GL, SA, and JW analyzed the data. JW, XL, MD, SA, and GL wrote the manuscript. JW, SA, and GL shared the microscopic observations and writing responsibilities. All authors have read and approved the manuscript for publication.

### Conflict of interest statement

The authors declare that the research was conducted in the absence of any commercial or financial relationships that could be construed as a potential conflict of interest.
