# Supplementary material for: Transcriptomic Responses to Different Cry1Ac Selection Stresses in Helicoverpa armigera
Source: Front Physiol. 2018 Nov 22;9:1653. doi: 10.3389/fphys.2018.01653 (PMC6262065; doi:10.3389/fphys.2018.01653)
Supplement: Supplementary file 1 [file Data_Sheet_1.PDF]

Transcriptomic responses to different Cry1Ac selection stresses in *Helicoverpa armigera*

Jizhen Wei[a,b], Shuo Yang[a], Lin Chen[b], Xiaoguang Liu [a], Mengfang Du [a], Shiheng An[a*], Gemei Liang[b*]

[a]State key Laboratory of Wheat and Maize Crop Science/College of Plant Protection, Henan Agricultural University, Zhengzhou 450002, China;

[b]State Key Laboratory for Biology of Plant Diseases and Insect Pests, Institute of Plant Protection, Chinese Academy of Agricultural Sciences, Beijing 100193, China;

[*]Author to whom correspondence should be addressed; E-mail: anshiheng@aliyun.com, gmliang@ippcaas.cn.

Table S1. Primers of candidate receptor genes used by quantitative RT-PCR

Name	Sequences(5'-3')
GADPH-RTF	CATTGAAGGTCTGATGACCACTGT
GADPH-RTR	CAGAGGGTCCATCCACTGTCTT
GADPH-RTP	CACGCCACCATTGCCACCCA
Actin-RTF	GGTGCACTGGCGATATTGG
Actin-RTR	CTTGGGTCTTGACAGCAATGC
Actin-RTP	AACCCCTTGGTCTGCCATGATAGCCTT
APN1-RTF	AGGTGGCAATTCGGTCATTAA
APN1-RTR	AGGATCAGCGAAGTTCTCTTCAA
APN1-RTP	ACGTATACTTTGAAACTGG
APN5-RTF	CCATCTTGGTGTCAGTCATCAAC
APN5-RTR	CTGGATGGCAGCGAAGTTCT
APN5-RTP	CCAGCCCCAACAGTACTCAGGCAGCTTTC
ALP-like-RTF	CCCGACGGACAGGTCATC
ALP-like-RTR	CCCTCCACGAACAGGAAGAA
ALP-like-RTP	ACGCAATGAGAAGGG
ALP2-RTF	ACACTAATGGACCCGGATTCC
ALP2-RTR	GCGATAGTTTGGTTCTGCAGTAAC
ALP2-RTP	CCACACGTGAATGAATGACATCCGGCA

Table S2. Primers of candidate trypsin genes used by quantitative qRT-PCR

Name	Sequences(5'-3')
18S-F	GCATCTTTCAAATGTCTGC
18S-R	TACTCATTCCGATTACGAG
EF-1α-F	GCCTGGTACCATTGTCGTCT
EF-1α-R	GTAACCACGACGCAACTCCT
XM_021340599-F	TGGCGTATCCGTCTTGG
XM_021340599-R	TGGTTGTCGGCGAGGTT
XM_021340597-F	ATTGTCGGTGGCTCCCTC
XM_021340597-R	AATGAGCAGCGGTTAGGA
XM_021329499-F	ATTGTAGGCGGCGTGGAG
XM_021329499-R	TTGACGAAGCAGTGAGCC
Trypsin-F	CTGGCAAGACTGTGGTGG
Trypsin -R	GGATACGGAATCGGTTGG
XM_021333008-F	AAGCACCCTGACTACACCG
XM_021333008-R	CCAACTAACGACCTGAACG
XM_021338512-F	TGTCTGCGGCTCACTGCTT
XM_021338512-R	AGGCGTGGTCAGCATCAGC
XM_021340600-F	CAACCTGAACGCCAACATC
XM_021340600-R	TTGTTGAAGACGAAGGTGG

Primers of trypsin were made to amplify a 95 bp conserved region among the three trypsin genes (accession no. XM_021344340.1, accession no. XM_021344341.1and accession no. XM_021344468.1).

Figure S1.

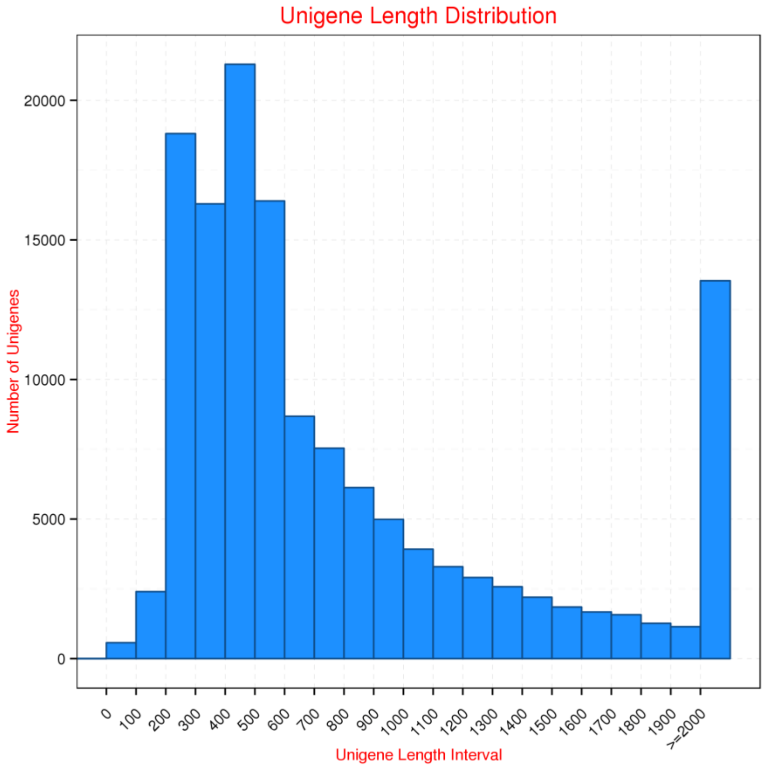

Figure S1. Unigene length distribution. These unigene were got from midgut samples of these seven strains and mixed with an unpublic data of cotton bollworm transcriptome database.

Figure S2.

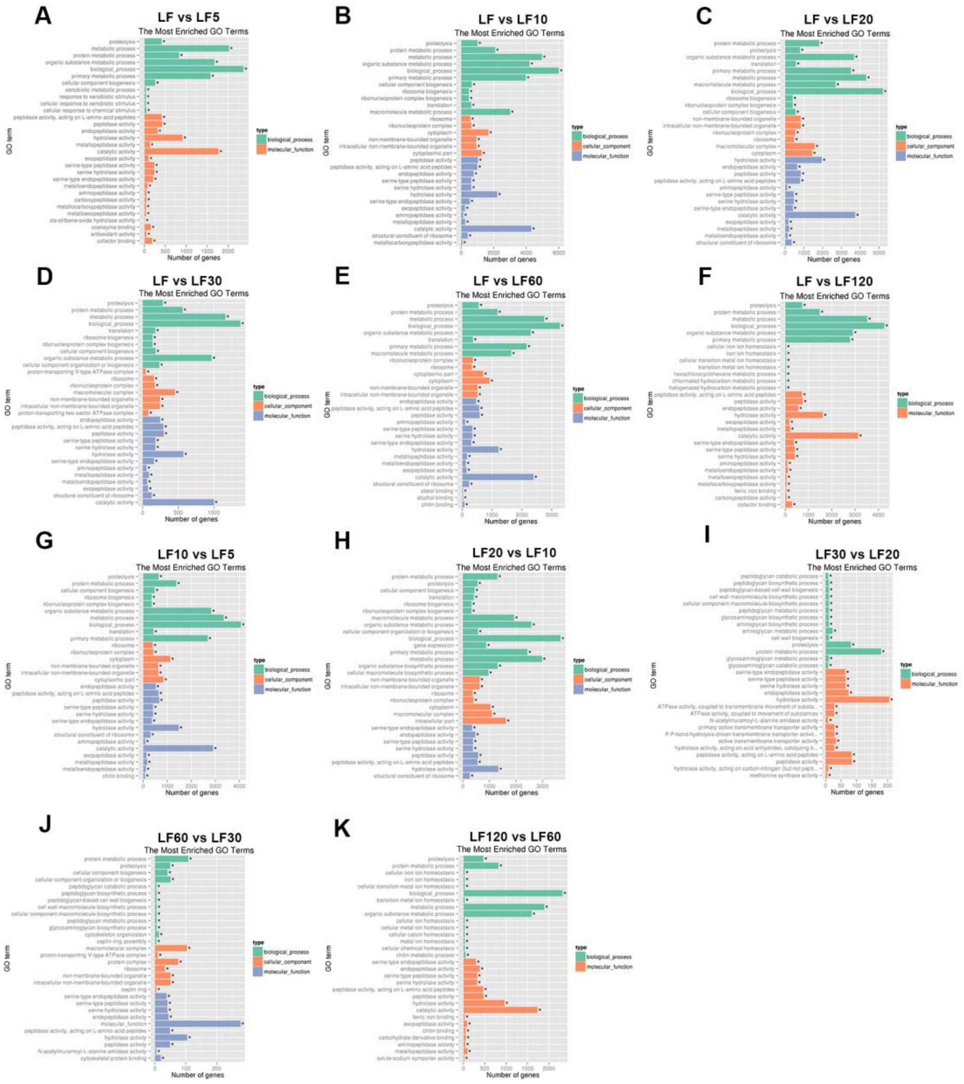

Figure S2. Gene ontology (GO) classification of different expression gene in LF and each of six LF resistant strans, two adjacent LF resistant strans. The Y-axis is the enrichment of the GO term; the The X-axis is the number of different expression among two different strains. Different colors were taken to distinguish biological process, cellular components and function of molecules.

Figure S3.

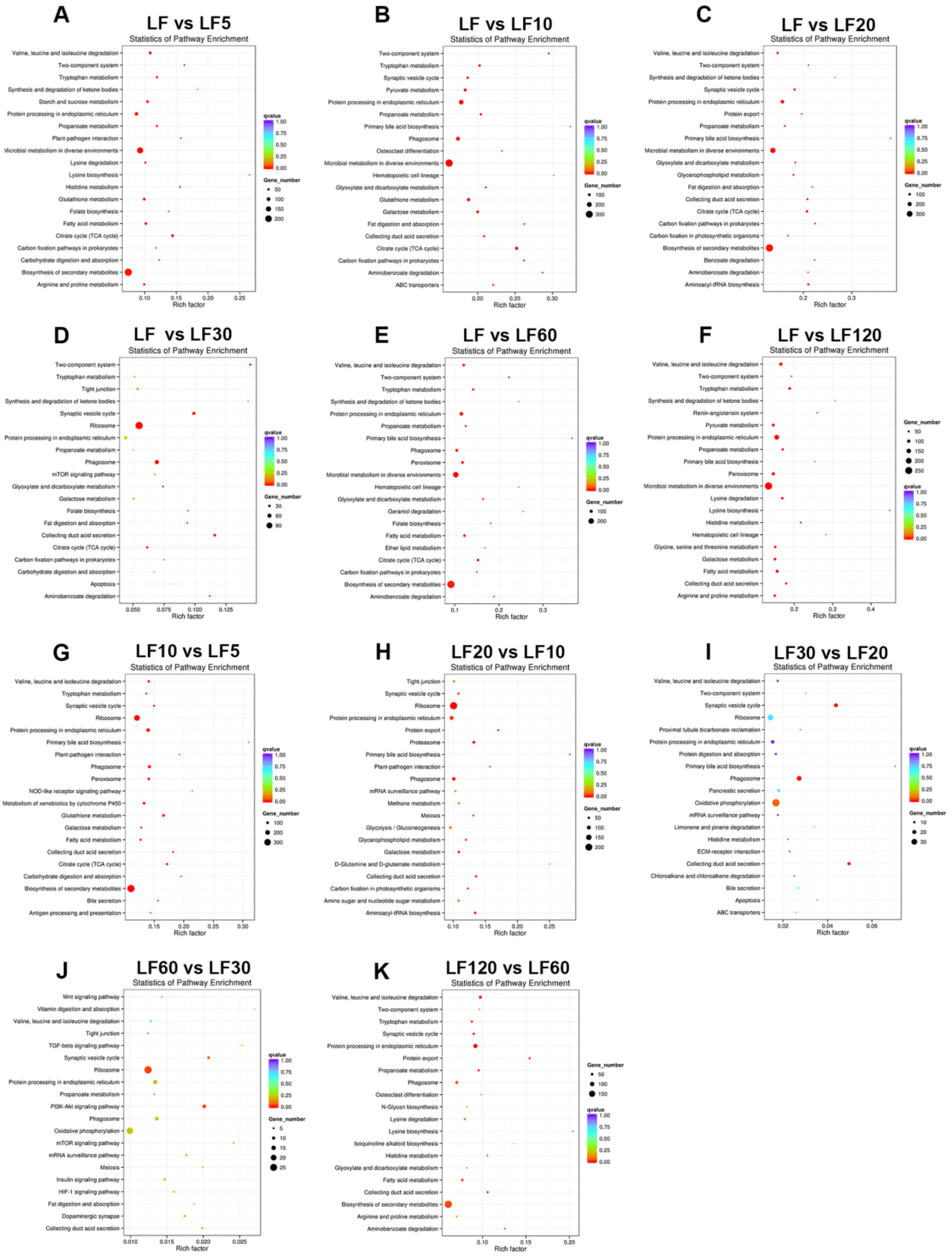

Figure S3. KEGG Ortholog classification of different expression gene in LF and each of six LF resistant strans, two adjacent LF resistant strans. The Y-axis is the pathway, X-axis shows rich factor, the size of the points according to how much the number of differentially expressed genes in this pathway, and the color of the points corresponding to different Qvalue range.
